# Musculoskeletal Images Classification for Detection of Fractures Using Transfer Learning

**DOI:** 10.3390/jimaging6110127

**Published:** 2020-11-23

**Authors:** Ibrahem Kandel, Mauro Castelli, Aleš Popovič

**Affiliations:** 1 Nova Information Management School (NOVA IMS), Universidade Nova de Lisboa, Campus de Campolide, 1070-312 Lisboa, Portugal; mcastelli@novaims.unl.pt (M.C.); ales.popovic@ef.uni-lj.si (A.P.); 2 School of Economics and Business, University of Ljubljana, Kardeljeva Ploščad 17, 1000 Ljubljana, Slovenia

**Keywords:** transfer learning, computer vision, convolutional neural networks, image classification, musculoskeletal images, deep learning, medical images

## Abstract

The classification of the musculoskeletal images can be very challenging, mostly when it is being done in the emergency room, where a decision must be made rapidly. The computer vision domain has gained increasing attention in recent years, due to its achievements in image classification. The convolutional neural network (CNN) is one of the latest computer vision algorithms that achieved state-of-the-art results. A CNN requires an enormous number of images to be adequately trained, and these are always scarce in the medical field. Transfer learning is a technique that is being used to train the CNN by using fewer images. In this paper, we study the appropriate method to classify musculoskeletal images by transfer learning and by training from scratch. We applied six state-of-the-art architectures and compared their performance with transfer learning and with a network trained from scratch. From our results, transfer learning did increase the model performance significantly, and, additionally, it made the model less prone to overfitting.

## 1. Introduction

Bone fractures are among the most common conditions that are treated in emergency rooms [1]. Bone fractures represent a severe condition that could result from an accident or a disease like osteoporosis. The fractures can lead to permanent damage or even death in severe cases. The most common way of detecting bone fractures is by investigating an X-ray image of the suspected organ. Reading an X-ray is a complex task, especially in emergency rooms, where the patient is usually in severe pain, and the fractures are not always visible to doctors. Musculoskeletal images are a subspecialty of radiology, which includes several techniques like X-ray, Computed Tomography (CT), and Magnetic Resonance Imaging (MRI), among others. For detecting fractures, the most commonly used method is the musculoskeletal X-ray image [2]. This process involves the radiologists, who are the doctors responsible for classifying the musculoskeletal images, and the emergency physicians, who are the doctors present in the emergency room where any patient with a sudden injury is admitted once arrived at the hospital. Emergency physicians are not very experienced in reading X-ray images like the radiologists, and they are prone to errors and misclassifications [3,4]. Image-classification software can help emergency physicians to accurately and rapidly diagnose a fracture [5], especially in emergency rooms, where a second opinion is much needed and, usually, is not available.

Deep learning is a recent breakthrough in the field of artificial intelligence, and it has demonstrated its potential in learning and prioritizing essential features of a given dataset without being explicitly programmed to do so. The autonomous behavior of deep learning makes it particularly suitable in the field of computer vision. The area of computer vision includes several tasks, like image segmentation, image detection, and image classification. Deep learning was successfully applied in many computer vision tasks, like in retinal image segmentation [6], histopathology image classification [7], and MRI image classification [8], among others. 

Focusing on image classification, in 2012, Krizhevsky et al. [9] proposed a convolutional neural network CNN-based model, and they won a very popular image classification challenge called ILSVRC. Afterward, CNNs gained popularity in the area of computer vision, and it is nowadays considered the state-of-the-art technique for image classification. The process of training a classifier is time-consuming and requires large datasets to be correctly trained. In the medical field, there is always a scarcity of images that can be used to train a classifier, mainly due to the regulations implemented in the medical field. Transfer learning is a technique that is usually used to train CNNs, when there are not enough images available or when obtaining new images is particularly difficult. Transfer learning is about training a CNN to classify large non-medical datasets and then use the weights of such a CNN as a starting point for classifying other target images, in our case, X-ray images. 

Several studies addressed the classification of musculoskeletal images using deep learning techniques. Rajpurkar et al. [10] introduced a novel dataset called MURA dataset that contains 40,005 musculoskeletal images. The authors used DenseNet169 CNN to compare the performance of the CNN against three radiologists. The model achieved an acceptable performance compared to the predictions of the radiologists. Chada [11] investigated the performance of three state-of-the-art CNNs, namely DenseNet169, DenseNet201, and InceptionResNetV2, on the MURA dataset. The author fine-tuned the three architectures using Adam optimizer with a learning rate of 0.0001. Fifty epochs were used with a batch size of eight images to train the model. The author reported that DenseNet201 achieved the best performance for the humerus images, with a Kappa score of 0.764, and InceptionResNetV2 achieved the best performance for the finger images, with a Kappa score of 0.555. 

To demonstrate the importance of deep learning in the emergency room for fracture detections, Lindsey et al. [5] investigated the usage of CNNs to detect wrist fractures. Subsequently, they measured the radiologists’ performance of detecting fractures with and without the help of CNN. The authors reported that, by using a CNN, the performance of the radiologists increased significantly. Kitamura et al. [12] studied the possibility of detecting ankle fractures with CNNs, using InceptionV3, ResNet, and Xception networks for their experiments. The authors trained a CNN from scratch without any transfer learning, and they used a private dataset and an ensemble of the three architectures and reported an accuracy of 81%.

In this paper, we are extending the work of Rajpurkar et al. [10] and Chada [11] by investigating the usage of transfer learning of a CNN to classify X-ray images to detect bone fractures. To do so, we used six state-of-the-art CNN architectures that were previously trained on the ImageNet dataset (an extensive non-medical dataset). To the best of our knowledge, this is the first paper that performs a rigorous investigation on the use of transfer learning in the context of musculoskeletal image classification. More in detail, we investigate the following:

1. The effect of transfer learning on image classification performance. To do that, we compare the performance of six CNN architectures that were trained on ImageNet to classify fractures images. Then, we train the same datasets with the same networks, but without the ImageNet weights.
2. The best classifier that achieves the best results on the musculoskeletal images.
3. The effect of the fully connected layers on the performance of the network. To do that, two fully connected layers were added after each network, and then we recorded their performance. Subsequently, the layers are removed, and the performance of the networks is recorded as well.

The paper is organized as follows: In Section 2, we present the methodology used. In Section 3, we present the results achieved by training the MURA dataset on the considered CNNs. In Section 4, we present a discussion about the results obtained, and we compare them to other state-of-the-art results. In Section 5, we conclude the paper by summarizing the main findings of this work.

## 2. Methodology

In this section, we briefly describe the main methods used in this paper. 

### 2.1. Convolutional Neural Networks and Transfer Learning

A convolutional neural network is a feed-forward neural network with at least one convolution layer. A convolution layer is a hidden neural network layer that has a convolution operation, where the convolution operation is a mathematical operation that is used to make use of the spatial information presented in images. Training a CNN requires a significant amount of images, and this is one of the most severe limitations in deep learning. In particular, deep learning has a very strong dependence on massive training data compared to traditional machine learning methods because it needs a large amount of data to understand the latent patterns of data. Unfortunately, there are problems in which insufficient training data are an inescapable issue. This may happen in domains in which obtaining new observations are either expensive, time-consuming, or impossible. In these situations, transfer learning provides a suitable way for training a CNN. More in detail, transfer learning is a technique used in the deep learning field to make use of knowledge that can be shared by different domains. According to Pan and Yang [13], transfer learning can be defined as improving the predictive function, $f_{T}\left(.\right)$ by using the knowledge acquired from the source domain, $D_{S}$, into the target domain, $D_{T}$. Transfer learning relaxes the hypothesis that the training data must be independent and identically distributed with the test data. This allows us to use transfer learning for training CNNs in a given domain and to use them to subsequently address a problem in which data scarcity is a significant limitation. For more details on transfer learning, the interested reader is referred to Pan and Yang [13].

### 2.2. State-of-the-Art Architectures

Many CNNs were introduced to participate in the ILSVRC challenge. In this section, we present different CNNs that are considered in this study.

#### 2.2.1. VGG

Simonyan et al. [14] introduced the VGG network in 2014. The VGG was implemented in many variations like VGG16 and VGG19, which only differ in the number of convolution layers used in each. In this paper, we use VGG19 because it is the largest one, and it usually produces better performance than VGG16. VGG19 consists of 19 convolution layers and one dense layer with 4096 neurons to classify the ImageNet images. For more information, the reader is referred to the corresponding paper [14]. 

#### 2.2.2. Xception

Chollet [15] introduced a novel architecture called Xception (extreme inception), where the author replaced the conventional convolutional layers with depthwise separable convolutional layers. These modified layers decreased the network parameters without decreasing its capacity, which yielded a robust network with fewer parameters and so less computational resources needed for training. For more information, the reader is referred to the corresponding paper [15].

#### 2.2.3. ResNet

ResNet architecture was introduced by He et al. [16] in 2015. ResNet was developed by exploiting the concept of residual connections. The authors introduced the concept of a residual connection to minimize the effect of the vanishing gradient. The ResNet architecture comes in many variants. In this paper, we use the ResNet50 network, which contains 50 layers. For more information, the reader is referred to the corresponding paper [16]. 

#### 2.2.4. GoogLeNet

GoogLeNet architecture was introduced by Szegedy et al. [17] in 2014. The authors proposed a novel idea called the inception module, which takes the aspect ratio of each image into account. There are many variants for GoogLeNet architecture, and we use the InceptionV3 network. For more information, the reader is referred to the corresponding paper [17]. 

#### 2.2.5. InceptionResNet

Längkvist et al. [18] created a novel architecture called InceptionResNet, where the authors combined the inception module idea from GoogLeNet architecture [17] with the residual idea from ResNet architecture [16]. InceptionResNet is more computationally efficient than both ResNet and Inception architectures and achieved higher results than both on the ImageNet dataset. For more information, the reader is referred to the corresponding paper [18]. 

#### 2.2.6. DenseNet

Huang et al. [19] introduced a novel architecture called DenseNet. In this architecture, the convolution blocks are densely connected to each other, and the convolution blocks are concatenated to each other instead of being added like in the ResNet network. For more information, the reader is referred to the corresponding paper [19].

### 2.3. Evaluation Metrics

Two evaluation metrics are being used to assess the performance of each network. Accuracy and Kappa. Below, we briefly summarize each metric:

#### 2.3.1. Accuracy

This metric quantifies how accurate the classifier is. It is calculated as the number of correctly classified data points divided by the total number of the data points. The formula is shown in Equation (1).

(1) $$\mathrm{Accuracy}=\frac{\mathrm{TN}+\mathrm{TP}}{\mathrm{TP}+\mathrm{TN}+\mathrm{FP}+\mathrm{FN}}$$

where TP stands for true positive, TN stands for true negative, FP stands for false positive, and FN stands for false negative. In the context of this study, images without fractures belong to the negative class, whereas images with a bone fracture belong to the positive class. 

#### 2.3.2. Kappa

This is an evaluation metric that is usually used to take into account the probability of selecting by chance, especially in cases of unbalanced datasets, and it was introduced by Cohen [20]. The upper limit of the Kappa metric is 1, which means that the classifier classified everything correctly. At the same time, the lower bound can go below zero, which indicates the classifier is just classifying by luck. The Kappa formula is presented in Equation (2).

(2) $$\mathrm{Kappa}=\frac{{\mathrm{Agreement}}_{\mathrm{Observed}}-{\mathrm{Agreement}}_{\mathrm{Expected}}}{1-{\;\mathrm{Agreement}}_{\mathrm{Expected}}}$$

### 2.4. Statistical Analysis

A statistical analysis was performed to assess the statistical significance of the results. We considered a confidence interval with a 95% error rate (95% CI) and a hypothesis test. Two hypothesis tests can be used: ANOVA or Kruskal–Wallis test. The choice of the test mainly depends on the normality of the data under observation. The ANOVA test is a parametric test that assumes that the data have a normal distribution. The null hypothesis of the ANOVA test is that the considered samples have the same mean, and the alternative hypothesis is that the samples have a different mean. 

The non-parametric test is the Kruskal–Wallis hypothesis test [21]. This test does not make any assumption on the normality of the data, and it compares the medians of different samples. The null and alternative hypotheses tested are the following:

**Hypothesis 0** **(H0).** *The populations medians are all equal.*

**Hypothesis 1** **(H1).** *The populations medians are not all equal.*

In this paper, we first tested the normality assumption by using the Shapiro–Wilk test. Considering that the test does not allow to reject the alternative hypothesis (i.e., data not normally distributed), the Kruskal–Wallis test was used to test the significance of the results obtained. To make the hypothesis test and to report means and significance errors, each setting was repeated 30 times using different seeds and different validation split. In this way, each approach’s stability can be assessed by also mitigating the effect of lucky seeds [22].

### 2.5. Dataset

The dataset used in this paper is the publicly available MURA dataset [10]. The dataset consists of seven different skeletal bones: elbow, finger, forearm, hand, humerus, shoulder, and wrist. Each category has a binary label, indicating if the image presents a broken bone or not. The dataset contains a total of 40,005 images. The authors of the dataset split it into training and test sets. The train set included 21,935 images without fractures (54.83% of the dataset) and 14,873 images with fractures (37.17% of the dataset), and the test set contained 1667 images without fractures (4.16% of the dataset) and 1530 images with fractures (3.84% of the dataset).

**Figure 1:**
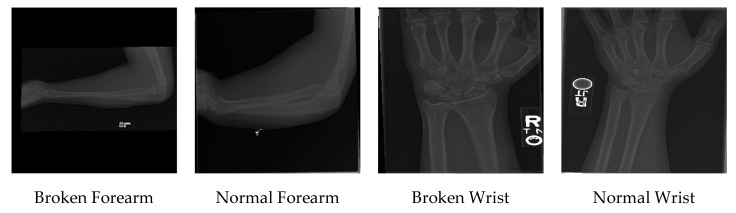
A sample of the MURA dataset.

All in all, 92% of the dataset is used for training, and 8% of the dataset is used for testing the results. The summary of the dataset is presented in Table 1. A sample of the MURA dataset is presented in Figure 1.

## 3. Results

Throughout the experiments, all the hyperparameters were fixed. All the networks were either fine-tuned completely or trained from scratch. Adam optimizer [21] was used in all the experiments. As noted by studies [22,23], the learning rate should be low to avoid dramatically changing the original weights, so we set the learning rate to be 0.0001. All the images were resized to 96×96 pixels. Binary cross-entropy was used as the loss function because the images are binary classified. An early stopping criterion of 50 epochs would be used to stop the algorithms if no updates happened to the validation score. The batch size was selected to be 64, and the training dataset was split into 80% to train and 20% to validate the results during training. Four image augmentation techniques were used to increase the training dataset’s size and make the network more robust against overfitting; the augmentation techniques used are horizontal and vertical flips, 180 rotations, and zooming.

Additionally, image augmentation is performed to balancing the number of images in the two target classes, thus achieving 50% of images without fractures and 50% of images with fractures in the training set. After the training, each network’s performance was tested using the dataset that was supplied by the owner and creator of the dataset. The test dataset was not used during the training phase, but only in the final testing phase. The hyperparameters used are presented in Table 2. In the following sections, Kappa is the metric considered for comparing the performance of the different architectures.

### 3.1. Wrist Images Classification Results 

Two main sets of experiments were performed: the first consists of adding two fully connected layers after each architecture to act as a classifier block. The second consists of adding only a sigmoid layer after the network. Both the results of the first set and the second set are presented in Table 3. 

In the first set of experiments, the fine-tuned VGG19 network had a Kappa score of 0.5989, while the network that was trained from scratch had a score of 0.5476. For the Xception network, the transfer learning score was higher than the one trained from scratch by a large margin. The ResNet50 network performance improved significantly by using transfer learning rather than training it from scratch. This indicates that transfer learning is fundamental for this network, that it could not learn the features of the images from scratch. Both the fine-tuned InceptionV3, InceptionResNetV2, and DenseNet121 networks have a higher score than training them from scratch. Overall, fine-tuning the networks did yield better results than training the networks from scratch. The best performance for the first set of experiments was achieved by fine-tuning the DenseNet121 network. 

In the second set of experiments, all the networks’ performance increased by fine-tuning than by training from scratch. The ResNet network was the network with the highest difference between fine-tuning and training from scratch. Overall, the best performance for the second set of experiments was achieved by fine-tuning the Xception network. Comparing the first set of experiments to the second set, we see that the best performance for classifying wrist images was the fine-tuned DenseNet121 network with fully connected layers. The presence of fully connected layers did not have any noticeable increase in performance; however, it is worth noting that the ResNet network with fully connected layers did not converge when trained from scratch. 

### 3.2. Hand Images Classification Results 

As done with the wrist images, two sets of experiments were performed. Both the results of the first set and the second set are presented in Table 4.

In the first set of experiments, for the VGG19 and the ResNet networks, fine-tuning the networks resulted in significantly higher performance than training the networks from scratch. The networks trained from scratch did not converge to an acceptable result. This fact highlights that the importance of transfer learning for these networks, that are not able to learn the images’ features from scratch. For the remaining networks, fine-tuning achieved significantly better performance than by training the networks from scratch. Overall, all the fine-tuned networks achieved better results than by training from scratch. The best performance of the first set of experiments was obtained with the fine-tuned Xception network.

In the second set of experiments, the performance of all the networks increased by fine-tuning than by training from scratch. The ResNet network was the network with the highest difference between fine-tuning and training from scratch. Overall, the best network was the VGG19 network. Comparing the first set of experiments to the second set, we see that the best performance for classifying hand images was the fine-tuned Xception network with fully connected layers. The presence of fully connected layers did not significantly increase the performance; however, it is important to point out that the VGG19 network with fully connected layers did not converge when it was trained from scratch.

### 3.3. Humerus Images Classification Results 

For the humerus images, the results of both the first and second sets of experiments are presented in Table 5. In the first set of experiments, fine-tuning VGG19 architecture did not converge to any acceptable results, while training the VGG19 from scratch did yield higher performance. For the rest of the networks, fine-tuning did achieve better results than training the networks from scratch. The highest difference was between fine-tuning the ResNet network and training it from scratch. Overall, the best network in the first sets of experiments was the fine-tuned DenseNet network, with a Kappa score of 0.6260. 

In the second set of experiments, fine-tuning did achieve better results for all the networks than training the networks from scratch. The best-achieved network was the VGG19 network, with a Kappa score of 0.6333. Comparing the first set of experiments to the second set, we see that the best performance for classifying humerus images was the fine-tuned VGG19 network without fully connected layers. Just as in the previous experiments, the fully connected layers’ presence did not provide any significant performance improvement; however, fine-tuning the VGG19 with fully connected layers did not converge compared to fine-tuning the same network without any fully connected layers. 

### 3.4. Elbow Images Classification Results

For the elbow images, we performed the same two sets of experiments performed with the previously analyzed datasets. Both the results of the first set and the second set are presented in Table 6. In the first set of experiments, the fine-tuned VGG19 score was less than training the same network from scratch. For the rest of the networks, fine-tuning did achieve higher performance than training the networks from scratch. The ResNet network achieved the highest difference between fine-tuning and training from scratch. Overall, the best network was the fine-tuned DenseNet121, with a Kappa score of 0.6510.

In the second set of experiments, no fully connected layers were added. For all the networks, fine-tuning did achieve higher results than training from scratch. Overall, the best network was the fine-tuned Xception network, with a Kappa score of 0.6711. Comparing the first set of experiments to the second set, we see that the best performance for classifying elbow images was the fine-tuned Xception network without fully connected layers.

### 3.5. Finger Images Classification Results

As with the previous datasets, two main sets of experiments were performed. Both the results of the first set and the second set are presented in Table 7. In the first set of experiments, fine-tuning achieved better results than training the networks from scratch for all the networks. The best-achieved network was the fine-tuned VGG19, with a Kappa score of 0.4379. In the second set of experiments, fine-tuning produced better results than training from scratch for all the six networks. The best network was the fine-tuned InceptionResNet network, with a Kappa score of 0.4455. Comparing the first set of experiments to the second set, we see that the best performance for classifying finger images was the fine-tuned InceptionResNet network without fully connected layers. Moreover, in this case, the presence of the fully connected layers did not provide any significant advantage in terms of performance.

### 3.6. Forearm Images Classification Results

As with the previous datasets, two sets of experiments were performed on the forearm images dataset. Both the results of the first set and the second set are presented in Table 8. In the first set of experiments, fine-tuning all the networks produced better results than training from scratch. Training of ResNet network from scratch did not yield any satisfactory results, which can imply that fine-tuning this network was crucial for obtaining a good result. The best network was the DenseNet121 network, with a Kappa score of 0.5851. Moreover, in the second set of experiments, fine-tuning achieved better results than training from scratch. The best network was the fine-tuned ResNet network, with a Kappa score of 0.5673. Comparing the first set of experiments to the second set, we see that the best performance for classifying forearm images was the fine-tuned DenseNet network with fully connected layers. As observed in other datasets, the presence of the fully connected layers did not have any significant advantage in terms of performance.

### 3.7. Shoulder Images Classification Results

In the first set of experiments, the VGG19 network did not converge to an acceptable result by using both methods. For the rest of the networks, fine-tuning the networks achieved better results than training the networks from scratch. The best network was the fine-tuned Xception network, with a Kappa score of 0.4543. Both the results of the first set and the second set are presented in Table 9. In the second set of experiments, training the ResNet network from scratch achieved slightly better results than fine-tuning. For the rest of the networks, fine-tuning achieved better results. The best network was the fine-tuned VGG19, with a Kappa score of 0.4502. Comparing the first set of experiments to the second set, we see that the best performance for classifying shoulder images was the fine-tuned Xception network with fully connected layers. The presence of the fully connected layers did not show any significant advantage in terms of performance. Anyhow, the VGG19 network with fully connected layers did not converge to any satisfactory result compared to the same network without any fully connected layers. 

### 3.8. Kruskal–Wallis Results

We applied the Kruskal–Wallis test to assess the statistical significance of different settings. The Kruskal–Wallis test yielded a *p*-value < 0.05 for all the results, which indicates to reject the null hypothesis that the settings have the same median and to accept the alternative hypothesis that there is a statistically significant difference between different settings (transfer learning “with and without fully connected layers” vs. training from scratch “with and without fully connected layers”). 

## 4. Discussion

In this paper, we compared the performance of fine-tuning on six state-of-the-art CNNs to classify musculoskeletal images. Training a CNN network from scratch can be very challenging, especially in the case of data scarcity. Transfer learning can help solve this problem by initiating the weights with values learned from a large dataset instead of initializing the weights from scratch. Musculoskeletal images play a fundamental role in classifying fractures. However, these images are always challenging to be analyzed, and a second opinion is often required, which will not always be available, especially in the emergency room. As pointed out by Lindsey et al. [5], the presence of an image classifier in the emergency room can significantly increase physicians’ performance in classifying fractures. 

For the first research question, about the effect of transfer learning, we noted that transfer learning produced better results than training the networks from scratch. For our second research question, the classifier that achieved the best result for wrist images was the fine-tuned DenseNet121 with fully connected layers; the classifier that achieved the best performance for elbow images was the fine-tuned Xception network without fully connected layers; for finger images, the best classifier was the fine-tuned InceptionResNetV2 network without fully connected layers; for forearm images, the best classifier was the fine-tuned DenseNet network with fully connected layers; for hand images, the best classifier was a fine-tuned Xception network with fully connected layers; the best classifier for humerus images was the fine-tuned VGG19 network without fully connected layers; finally, the best classifier for classifying the shoulder images was the fine-tuned Xception network with fully connected layers. A summary of the best CNNs is presented in Table 10. Concerning the third research question, the fully connected layers had a negative effect on the performance of the considered CNNs. In particular, in many cases, it decreased the performance of the network. Further research is needed to study, in more detail, the impact of fully connected layers, especially in the case of transfer learning. 

The authors of the MURA dataset [10] assessed the performance of three radiologists on the dataset and compared their performance against the one of a CNN. In Table 11, we present their results, along with our best scores. 

For classifying elbow images, the first radiologist achieved the best score, and our score was comparable to other radiologists [10]. For finger images, our score was higher than the three radiologists. For forearm images, our score was lower than the radiologists. For hand images, our score was the lowest. For humerus images, shoulder images, and wrist images, our score was lower than the radiologists. We still believe that the scores achieved in this paper are promising, keeping in mind that these scores came from off-the-shelf models that were not designed for medical images in the first place and that the images were resized to be 96×96 pixels due to hardware limitations. Nevertheless, additional efforts are needed to outperform the performance of experienced radiologists.

On the other side of the spectrum, there is the study of Raghu et al. [24], where the authors argued that transfer learning is not good enough for medical images and will be less accurate compared to training from scratch or compared to novel networks explicitly designed for the problem at hand. The authors studied the effect of transfer learning on two medical datasets, namely, retina images and chest X-ray images. The authors stated that designing a lightweight CNN can be more accurate than using transfer learning. In our study, we did not consider “small” CNNs trained from scratch. Thus, it is not possible to directly compare the results obtained to the ones presented in Raghu et al. [24]. Anyway, more studies are needed to better understand the effect of transfer learning on medical-image classification. 

## 5. Conclusions

In this paper, we investigated the effect of transfer learning on classifying musculoskeletal images. We find that, out of the 168 results obtained that were performed by using six different CNN architectures and seven different bone types, transfer learning achieved better results than training a CNN from scratch. Only in 3 out of the 168 results did training from scratch achieve slightly better results than transfer learning. The weaker performance of the training-from-scratch approach could be related to the number of images in the considered dataset, as well as to the choice of the hyperparameters. In particular, the CNNs taken into account are characterized by the presence of a large number of trainable parameters (i.e., weights), and the number of images used to train these networks is too small to build a robust model. Concerning the hyperparameters, we highlight the importance of the learning rate. While we used a small value of the learning rate in the fine-tuning approach, to avoid changing the architectures’ original weights dramatically, the training-from-scratch approach could require a higher value of the learning rate. A complete study on the hyperparameters’ effect will be considered in future work, aiming to fully understand the best approach to be used when dealing with fracture images. Focusing on this study’s results, it is possible to state that transfer learning is recommended in the context of fracture images. In our future work, we plan to introduce a novel CNN to classify musculoskeletal images, aiming at outperforming fine-tuned CNNs. This would be the first step towards the design of a CNN-based system, which classifies the image and provides the probable position of the fracture if the fracture is present in the image.

## Figures and Tables

**Table 1:** MURA dataset summary.

Category	Training Dataset		Test Dataset	
	Normal	Fractured	Normal	Fractured
Elbow	2925	2006	235	230
Finger	3138	1968	214	247
Hand	4059	1484	271	189
Humerus	673	599	148	140
Forearm	1164	661	150	151
Shoulder	4211	4168	285	278
Wrist	5765	3987	364	295
Total	21,935	14,873	1667	1530

**Table 2:** The hyperparameters were used for all the experiments.

**Framework**	Keras with Python
**Optimizer**	Adam
**Learning Rate**	0.0001
**Loss Function**	Binary Cross-entropy
**Early Stopping**	50 epochs
**Batch Size**	64
**Validation Split**	20%
**Image Augmentation**	Horizontal flips
	Vertical flips
	180 rotations
	Zooming

**Table 3:** Accuracy and Kappa scores of classifying wrist images with and without fully connected layers (±95% CI).

		With FC		Without FC	
Network	Method	Mean Accuracy	Mean Kappa	Mean Accuracy	Mean Kappa
VGG19	TL	80.45% ± 1.26%	0.5989 ± 2.39%	80.63% ± 1.64%	0.6035 ± 3.33%
	Scratch	78.07% ± 0.94%	0.5476 ± 2.11%	79.89% ± 1.84%	0.5846 ± 3.74%
InceptionV3	TL	79.92% ± 2.07%	0.5886 ± 3.61%	79.94% ± 1.46%	0.5876 ± 2.77%
	Scratch	77.01% ± 2.98%	0.5241 ± 6.49%	77.59% ± 1.51%	0.5389 ± 3.03%
ResNet	TL	78.76% ± 0.88%	0.5647 ± 2.04%	80.85% ± 1.70%	0.6046 ± 3.63%
	Scratch	58.65% ± 8.70%	0.0836 ± 21.31%	70.99% ± 4.20%	0.4018 ± 8.27%
Xception	TL	80.93% ± 0.88%	0.6098 ± 1.69%	81.18% ± 0.47%	0.6133 ± 0.94%
	Scratch	77.44% ± 1.99%	0.5333 ± 4.41%	77.14% ± 1.86%	0.5318 ± 3.32%
DenseNet	TL	81.71% ± 0.94%	0.6245 ± 1.98%	78.76% ± 2.27%	0.5663 ± 4.29%
	Scratch	76.40% ± 2.30%	0.5083 ± 5.17%	76.68% ± 3.62%	0.5214 ± 7.25%
InceptionResNet	TL	80.1% ± 1.66%	0.5917 ± 3.32%	80.55% ± 1.06%	0.6010 ± 2.25%
	Scratch	77.77% ± 1.59%	0.5450 ± 2.85%	78.55% ± 1.70%	0.5580 ± 3.48%

**Table 4:** Accuracy and Kappa scores of classifying hand images with and without fully connected layers (±95% CI).

		With FC		Without FC	
Network	Method	Mean Accuracy	Mean Kappa	Mean Accuracy	Mean Kappa
VGG19	TL	70.11% ± 9.23%	0.3089 ± 25.42%	73.04% ± 1.27%	0.3960 ± 3.23%
	Scratch	58.91% ± 0%	0 ± 0%	63.22% ± 5.90%	0.1312 ± 15.90%
InceptionV3	TL	70.25% ± 1.34%	0.3261 ± 3.57%	72.10% ± 0.79%	0.3829 ± 2.15%
	Scratch	66.38% ± 3.80%	0.2382 ± 9.76%	66.56% ± 2.48%	0.2361 ± 6.07%
ResNet	TL	72.25% ± 1.25%	0.3754 ± 3.55%	71.12% ± 1.94%	0.3503 ± 5.44%
	Scratch	59.28% ± 0.93%	0.0103 ± 2.65%	62.10% ± 1.21%	0.0971 ± 3.26%
Xception	TL	75.36% ± 2.56%	0.4621 ± 6.27%	72.50% ± 2.0%	0.3778 ± 5.41%
	Scratch	66.74% ± 2.58%	0.2277 ± 7.20%	66.81% ± 3.60%	0.2334 ± 10.05%
DenseNet	TL	72.21% ± 1.69%	0.3746 ± 4.33%	70.22% ± 3.28%	0.3243 ± 9.03%
	Scratch	63.33% ± 1.16%	0.1308 ± 3.47%	62.79% ± 1.50%	0.1231 ± 4.39%
InceptionResNet	TL	71.96% ± 1.80%	0.3709 ± 4.33%	71.81% ± 1.91%	0.3670 ± 4.65%
	Scratch	68.48% ± 1.53%	0.2788 ± 4.36%	69.09% ± 1.04%	0.3071 ± 2.43%

**Table 5:** Accuracy and Kappa scores of classifying humerus images with and without fully connected layers (±95% CI).

		With FC		Without FC	
Network	Method	Mean Accuracy	Mean Kappa	Mean Accuracy	Mean Kappa
VGG19	TL	51.39% ± 0%	0 ± 0%	81.66% ± 2.74%	0.6333 ± 5.43%
	Scratch	62.04% ± 3.67%	0.239 ± 8.01%	69.44% ± 8.28%	0.3893 ± 16.77%
InceptionV3	TL	80.32% ± 2.74%	0.6070 ± 5.51%	80.56% ± 1.48%	0.6114 ± 2.94%
	Scratch	67.77% ± 3.12%	0.3603 ± 6.08%	64.06% ± 3.51%	0.2879 ± 6.81%
ResNet	TL	80.38% ± 2.55%	0.6084 ± 5.03%	78.18% ± 2.20%	0.5647 ± 4.31%
	Scratch	54.28% ± 7.46%	0.0849 ± 14.97%	65.63% ± 5.19%	0.3171 ± 10.26%
Xception	TL	80.03% ± 1.92%	0.6010 ± 3.81%	79.75% ± 1.67%	0.5942 ± 3.40%
	Scratch	66.55% ± 2.84%	0.3386 ± 5.46%	66.32% ± 4.72%	0.3334 ± 9.11%
DenseNet	TL	81.31% ± 1.88%	0.6260 ± 3.81%	77.84% ± 1.52%	0.5563 ± 3.16%
	Scratch	70.54% ± 5.85%	0.4134 ± 11.37%	71.93% ± 2.74%	0.4406 ± 5.35%
InceptionResNet	TL	78.41% ± 1.84%	0.5697 ± 3.61%	78.76% ± 2.56%	0.5761 ± 5.07%
	Scratch	65.34% ± 3.89%	0.3135 ± 7.61%	65.34% ± 4.37%	0.3139 ± 8.47%

**Table 6:** Accuracy and Kappa scores of classifying elbow images with and without fully connected layers (±95% CI).

		With FC		Without FC	
Network	Method	Mean Accuracy	Mean Kappa	Mean Accuracy	Mean Kappa
VGG19	TL	71.36% ± 16.95%	0.4232 ± 13.01%	81.61% ± 1.56%	0.6316 ± 3.12%
	Scratch	75.81% ± 34.45%	0.5136 ± 26.45%	76.52% ± 13.45%	0.5279 ± 27.33%
InceptionV3	TL	81.72% ± 0.91%	0.6339 ± 1.84%	80.93% ± 2.3%	0.6180 ± 4.6%
	Scratch	77.96% ± 2.92%	0.5583 ± 5.84%	76.09% ± 4.29%	0.5208 ± 8.6%
ResNet	TL	81.79% ± 3.28%	0.6351 ± 6.61%	81.9% ± 2.22%	0.6374 ± 4.44%
	Scratch	56.20% ± 9.60%	0.1161 ± 19.68%	71.04% ± 4.02%	0.4191 ± 8.09%
Xception	TL	82.15% ± 1.21%	0.6425 ± 2.42%	83.58% ± 1.64%	0.6711 ± 3.31%
	Scratch	78.21% ± 2.83%	0.5631 ± 5.70%	78.49% ± 1.88%	0.5690 ± 3.75%
DenseNet	TL	82.58% ± 1.97%	0.6510 ± 3.92%	81.08% ± 2.23%	0.6208 ± 4.47%
	Scratch	75.38% ± 3.36%	0.5060 ± 6.82%	73.84% ± 4.70%	0.4754 ± 9.49%
InceptionResNet	TL	80.82% ± 0.61%	0.6159 ± 1.21%	80.47% ± 1.68%	0.6087 ± 3.37%
	Scratch	79.82% ± 1.45%	0.5955 ± 2.91%	78.49% ± 1.28%	0.5694 ± 2.58%

**Table 7:** Accuracy and Kappa scores of classifying finger images with and without fully connected layers (±95% CI).

		With FC		Without FC	
Network	Method	Mean Accuracy	Mean Kappa	Mean Accuracy	Mean Kappa
VGG19	TL	71.4% ± 1.84%	0.4379 ± 3.59%	68.44% ± 2.76%	0.3847 ± 5.10%
	Scratch	66.78% ± 2.83%	0.3505 ± 5.22%	66.16% ± 3.10%	0.3413 ± 5.26%
InceptionV3	TL	67.68% ± 2.15%	0.3686 ± 3.87%	68.55% ± 3.19%	0.3834 ± 5.97%
	Scratch	63.52% ± 2.66%	0.2911 ± 4.82%	63.88% ± 2.15%	0.2916 ± 4.09%
ResNet	TL	70.17% ± 1.16%	0.4129 ± 2.17%	69.02% ± 2.52%	0.3900 ± 4.44%
	Scratch	60.34% ± 8.07%	0.2341 ±13.90%	66.41% ± 2.98%	0.3431 ± 5.68%
Xception	TL	71.37% ± 2.43%	0.4369 ± 4.42%	70.64% ± 2.27%	0.4234 ± 4.28%
	Scratch	64.75% ± 2.79%	0.3109 ± 5.33%	64.57% ± 2.72%	0.3055 ± 5.31%
DenseNet	TL	66.78% ± 2.64%	0.3552 ± 4.66%	66.81% ± 1.92%	0.3512 ± 3.61%
	Scratch	62.18% ± 2.10%	0.2692 ± 3.60%	64.32% ± 3.16%	0.3051 ± 5.64%
InceptionResNet	TL	70.97% ± 2.05%	0.4294 ± 3.80%	71.8% ± 1.49%	0.4455 ± 2.88%
	Scratch	64.64% ± 3.07%	0.3112 ± 5.70%	65.29% ± 3.09%	0.3204 ± 5.72%

**Table 8:** Accuracy and Kappa scores of classifying forearm images with and without fully connected layers (±95% CI).

		With FC		Without FC	
Network	Method	Mean Accuracy	Mean Kappa	Mean Accuracy	Mean Kappa
VGG19	TL	77.02% ± 1.27%	0.5408 ± 2.54%	76.3% ± 2.16%	0.5264 ± 4.31%
	Scratch	64.29% ± 9.50%	0.2870 ± 18.91%	71.15% ± 6.41%	0.4237 ± 12.75%
InceptionV3	TL	76.52% ± 1.46%	0.5308 ± 2.91%	77.46% ± 1%	0.5496 ± 1.99%
	Scratch	64.84% ± 5.50%	0.2973 ± 11.01%	65.84% ± 4.95%	0.3171 ± 9.89%
ResNet	TL	74.7% ± 1.20%	0.4943 ± 2.38%	78.35% ± 3.46%	0.5673 ± 6.92%
	Scratch	50.11% ± 0.56%	0.0055 ± 1.11%	63.79% ± 3.58%	0.2764 ± 7.12%
Xception	TL	75.08% ± 1.84%	0.5022 ± 3.69%	76.08% ± 1.66%	0.5222 ± 3.32%
	Scratch	66.00% ± 2.54%	0.3204 ± 5.08%	65.73% ± 5.31%	0.3148 ± 10.64%
DenseNet	TL	79.24% ± 0.53%	0.5851 ± 1.05%	76.14% ± 2.32%	0.5232 ± 4.63%
	Scratch	68.77% ± 3.75%	0.3755 ± 7.51%	69.66% ± 3.12%	0.3935 ± 6.25%
InceptionResNet	TL	74.7% ± 2.64%	0.4945 ± 5.27%	74.86% ± 1.98%	0.4977 ± 3.95%
	Scratch	65.45% ± 3.36%	0.3096 ± 6.69%	69.1% ± 6.07%	0.3824 ± 12.12%

**Table 9:** Accuracy and Kappa scores of classifying shoulder images with and without fully connected layers (±95% CI).

		With FC		Without FC	
Network	Method	Mean Accuracy	Mean Kappa	Mean Accuracy	Mean Kappa
VGG19	TL	77.02% ± 1.27%	0.5408 ± 2.54%	76.3% ± 2.16%	0.5264 ± 4.31%
	Scratch	64.29% ± 9.50%	0.2870 ± 18.91%	71.15% ± 6.41%	0.4237 ± 12.75%
InceptionV3	TL	76.52% ± 1.46%	0.5308 ± 2.91%	77.46% ± 1%	0.5496 ± 1.99%
	Scratch	64.84% ± 5.50%	0.2973 ± 11.01%	65.84% ± 4.95%	0.3171 ± 9.89%
ResNet	TL	74.7% ± 1.20%	0.4943 ± 2.38%	78.35% ± 3.46%	0.5673 ± 6.92%
	Scratch	50.11% ± 0.56%	0.0055 ± 1.11%	63.79% ± 3.58%	0.2764 ± 7.12%
Xception	TL	75.08% ± 1.84%	0.5022 ± 3.69%	76.08% ± 1.66%	0.5222 ± 3.32%
	Scratch	66.00% ± 2.54%	0.3204 ± 5.08%	65.73% ± 5.31%	0.3148 ± 10.64%
DenseNet	TL	79.24% ± 0.53%	0.5851 ± 1.05%	76.14% ± 2.32%	0.5232 ± 4.63%
	Scratch	68.77% ± 3.75%	0.3755 ± 7.51%	69.66% ± 3.12%	0.3935 ± 6.25%
InceptionResNet	TL	74.7% ± 2.64%	0.4945 ± 5.27%	74.86% ± 1.98%	0.4977 ± 3.95%
	Scratch	65.45% ± 3.36%	0.3096 ± 6.69%	69.1% ± 6.07%	0.3824 ± 12.12%

**Table 10:** The best convolutional neural network (CNN) for each image category.

Fracture	CNN
Wrist	DenseNet
Elbow	Xception
Finger	InceptionResNetV2
Forearm	DenseNet121
Hand	Xception
Humerus	VGG19
Shoulder	Xception

**Table 11:** Kappa scores of three radiologists reported in Reference [10] compared to our results.

Fracture	1st Radiologist	2nd Radiologist	3rd Radiologist	Our Score
Elbow	0.850	0.710	0.719	0.671
Finger	0.304	0.403	0.410	0.445
Forearm	0.796	0.802	0.798	0.585
Hand	0.661	0.927	0.789	0.462
Humerus	0.867	0.733	0.933	0.633
Shoulder	0.864	0.791	0.864	0.454
Wrist	0.791	0.931	0.931	0.625
